# Nomenclature of Vertebral Laminae in Lizards, with Comments on Ontogenetic and Serial Variation in Lacertini (Squamata, Lacertidae)

**DOI:** 10.1371/journal.pone.0149445

**Published:** 2016-02-23

**Authors:** Emanuel Tschopp

**Affiliations:** 1 Dipartimento di Scienze della Terra, Università di Torino, Torino, Italy; 2 GeoBioTec, Faculdade de Ciências e Tecnologia (FCT), Universidade Nova de Lisboa, Caparica, Portugal; 3 Museu da Lourinhã, Lourinhã, Portugal; New York Institute of Technology College of Osteopathic Medicine, UNITED STATES

## Abstract

Vertebral laminae are bony ridges or sheets that connect important morphological landmarks on the vertebrae, like diapophyses or zygapophyses. They usually exhibit some serial variation throughout the column. A consistent terminology facilitates the morphological description of this variation, and the recognition of patterns that could be taxonomically significant and could serve as phylogenetic characters. Such a terminology was designed for saurischian dinosaurs, and has also been applied to other members of Archosauriformes. Herein, this terminology is applied for the first time to lizards (Squamata). Probably due to their generally smaller size compared to saurischian dinosaurs, lizards have less developed vertebral laminae. Some laminae could not be recognized in this group and others require new names to account for differences in basic vertebral morphology. For instance, the fusion of diapophysis and parapophysis in lacertids into a structure called synapophysis necessitates the creation of the new term synapophyseal laminae for both diapophyseal and parapophyseal laminae. An assessment of occurrence and serial variation in a number of lacertid species shows that some laminae develop throughout ontogeny or only occur in large-sized species, whereas the distribution of other laminae might prove to be taxonomically significant in future.

## Introduction

The vertebral column consists of presacral, sacral, and caudal elements. Presacral vertebrae of amniotes are generally subdivided into cervical and dorsal elements. Among reptiles, the number of the elements per subregion can vary considerably [1–3], to a small degree even intraspecifically (mostly due to sexual dimorphism [4–6]). Whereas 24 presacral vertebrae seems to be the basal count for reptiles, most squamate clades increased this number, in particular the legless snakes and amphisbaenians [1,2]. Lacertids generally have approximately 26 presacral vertebrae, ranging from 23 to 30 [1,6]. Usually, eight of these are cervical vertebrae [1,4], although rare exceptions exist [7]. Presacral vertebrae are followed by two sacral vertebrae, and a varying number of caudal elements [1,4,8].

Cervical vertebrae are usually defined as the elements anterior to the first vertebra connected with the sternum through a rib [1,5,9,10]. Rib morphology is also used to subdivide dorsal vertebrae into thoracic and lumbar elements [2,7,11]. Morphometric studies even revealed three distinct dorsal subregions in both terrestrial and aquatic *Varanus* [12].

Caudal vertebrae of lacertids are characterized by the ability of losing part of the tail (autotomy; see e.g. [8]). The caudal column can be subdivided in anterior, non-autotomic elements, anterior autotomic elements with double transverse processes, middle autotomic vertebrae with single transverse processes, and posterior, autotomic vertebrae without transverse processes [8]. All these subregions of the presacral, sacral, and caudal vertebral column have their own topographical and functional requirements [1,3], and particular changes in number of elements or morphology throughout the series have been proposed to be taxonomically significant in certain lizard taxa [5,8,13]. However, how the shape of the vertebrae in their entirety changes throughout the vertebral column of reptiles has rarely been assessed in detail (e.g. [3,5,12,14–16]).

Within Squamata, and in lacertids in particular, descriptions of the vertebral column are scarce, and usually concern general morphology in large taxonomic groups ([1,8]; see [11,17] for exceptions outside Lacertidae). The scarcity of morphological information might in part be due to a limited nomenclature of vertebral features and thus a greater difficulty to produce concise, detailed descriptions. The lack thereof results in low numbers of axial characters in phylogenetic analyses (e.g. three out of 64 morphological characters in [6]), and hampers the identification of fossils. In fact, fossil lacertid vertebrae are often only referred to Lacertidae indet. (e.g. [18,19]). In order to facilitate detailed vertebral descriptions and recognition of diagnostic morphological traits, I herein adapt the terminology of vertebral laminae developed for saurischian dinosaurs [20] to lizards.

Vertebral laminae have been mostly recognized in saurischian dinosaurs, where they are particularly developed in sauropods [20,21]. Given their highly diversified structures throughout the various sauropod clades, a nomenclature was a necessary, and well-received tool for systematic research: in recent phylogenetic analyses of titanosauriform and diplodocid sauropods, 13 and 24 percent of the analyzed axial characters mention variability in vertebral lamination (16 and 56 characters, respectively [22,23]). The initial proposal of a consistent terminology by Wilson [20] was further developed and adapted by several subsequent studies (e.g. [21,24,25]). Development and differentiation of vertebral lamination considerably changes throughout the column [3,20,21]. Given that the terminology for these laminae is landmark-based [20,21], the names have also been applied to more distantly related taxa, where the same landmarks could be identified [26–28]. It has to be noted, though, that this terminological system does not imply homology of structures with the same name [20].

Whereas general squamate phylogeny is slowly approaching some consent about the placement of major lineages [29–31], these large-scale studies do not provide much information concerning lacertid intrarelationships. The last detailed phylogenetic analysis at a lower taxonomic level was the one of Arnold et al. [6] concerning Lacertini. However, other than recognizing genus-level clades, Arnold et al. [6] were not able to resolve the relationships between these genera. This issue might in part be due to the limited number of morphological characters, in particular from the postcranial skeleton. The adaption of the terminology of Wilson [20] will facilitate the recognition of potentially useful axial characters in lacertids (and potentially other lizards), and provide the base for detailed descriptions of axial morphology. In order to demonstrate the utility of such a nomenclatural system I further assess serial variation and ontogenetic changes of the vertebral laminae in Lacertini.

## Material & Methods

### Ethics statement

No permits were required for this study, because the analyses were exclusively carried out on skeletal preparations deposited in the collections of the Museum of Geology and Paleontology of the Department of Earth Sciences of the University of Turin, Italy. No additional specimens were collected for this study.

### Material

The specimens at the University of Turin are cataloged under the acronym MDHC. Ten specimens are preserved with the articulated presacral column, where a string was passed through the neural canal during preparation, before disarticulation of the vertebrae.

The main part of this study is based on 20 lacertid specimens belonging to eleven species in seven genera. The specimens were chosen in order to cover species of varying body size and skeletally immature and mature individuals (Table 1). Seven lacertid specimens are preserved with articulated presacral, sacral, and anterior caudal vertebral columns. Four additional specimens representing the other major clades of lizards (Gekkota, Scincoidea, Anguimorpha, Iguania [31]) were studied in order to assess the utility of the adapted nomenclatural system among lizards in general (Table 1). Snakes were excluded because their derived morphology would merit its own, detailed assessment. However, having similar principal vertebral landmarks as other squamates, it is to be expected that the nomenclatural system can also be applied to Serpentes.

**Table 1:** Species and specimens examined.

Subclade	Species	Specimen	Articulated column	Skeletally immature	SVL (mm)
Gekkota	*Tarentola mauritanica*	MDHC 194	x		75
Scincoidea	*Chalcides ocellatus*	MDHC 193	x		155
Anguimorpha	*Varanus exanthematicus*	MDHC 335	x		not recorded
Iguania	*Agama impalearis*	MDHC 275			103
Lacertoidea	*Anatololacerta danfordi*	MDHC 283			69
		MDHC 284			69
	*Lacerta agilis*	MDHC 176	x		77
		MDHC 177	x		71
		MDHC 178	x		70
	*Lacerta bilineata*	MDHC 15	x		110
		MDHC 48		x	55
		MDHC 73		x	53
		MDHC 77	x		103
	*Lacerta strigata*	MDHC 304			not recorded
	*Lacerta trilineata*	MDHC 240			113
		MDHC 241			ca. 105
		MDHC 356			not recorded
	*Phoenicolacerta troodica*	MDHC 318			not recorded
		MDHC 319		x	not recorded
	*Podarcis muralis*	MDHC 313			incomplete
	*Podarcis wagleriana*	MDHC 390			not recorded
	*Takydromus* sp.	MDHC 151	x		58
	*Timon lepidus*	MDHC 216			150
	*Zootoca vivipara*	MDHC 179	x		59

### Methods

Occurrence and serial variation of the laminae was tracked under a light microscope. All vertebrae of the seven lacertid specimens with articulated vertebral column were observed to assess serial variation in detail. Of the non-lacertid lizards, and the disarticulated material, two cervical, two dorsal, both sacral, and an anterior, non-autotomic, and two autotomic caudal vertebrae were checked for occurrence of laminae.

Ontogenetic changes were recorded in three immature specimens of *Phoenicolacerta troodica* (MDHC 319) and *Lacerta bilineata* (MDHC 48, 73). Immaturity was established based on completely or partially unfused vertebral synchondroses: in one specimen of *L*. *bilineata* (MDHC 73), no neural arch was fused to the vertebral centrum, and only few had fused right and left halves of the neural arch. MDHC 73 can thus be considered the most immature specimen.

Serial photographs were taken with a digital camera applied to a Leica M205C stereomicroscope. The pictures were subsequently processed with the Leica Application Suite V. 3.3. in order to avoid focal distortion and a short depth of field.

The figures of the presacral columns in the supporting information tables illustrating serial variation were produced with the freeware VertFigure (Copyright 2013–2014, Mike Taylor mike@miketaylor.org.uk). The software is described and available for free through the blog Sauropod Vertebra Picture of the Week: http://svpow.com/2014/04/12/introducing-vertfigure-a-better-name-for-vcd2svg/.

## Terminology

The basic nomenclature proposed by Wilson [20] defines laminae as ridges or sheets of bone that connect two well-established vertebral landmarks. These landmarks mainly include the centrum, the diapophysis, the parapophysis, the zygapophyses, and the neural spine. Wilson [20] arbitrarily assigned preferences of certain landmarks over others, in order to maintain consistency of the nomenclature. For instance, the diapophysis has preference over the prezygapophysis, therefore a lamina connecting the two should be called prezygodiapophyseal lamina [20]. A unique four letter acronym is then assigned to the lamina, recalling first the secondary and then the primary landmarks. In the case of the prezygodiapophyseal lamina, this would be PRDL [20].

An adaptation of the nomenclature designed for saurischian dinosaurs to lizards obviously requires certain changes. Increased vertebral lamination in sauropod dinosaurs is correlated with large body size and neck elongation [32]. Given the much smaller size of lizards, lamination patterns are less developed, and vertebral shape in general is simplified compared to sauropods. Therefore, many of the laminae in sauropods do not occur in lizards, and some of the names proposed by Wilson [20] have to be adapted. In total, 13 vertebral laminae are counted in lizards (Tables 2, 3).

**Table 2:** Vertebral laminae present in non-lacertid Squamata, showing occurrence in presacral, sacral, and caudal vertebrae.

Higher-level taxon		Gekkota			Scincoidea			Anguimorpha			Iguania		
Species		*Tarentola mauritanica*			*Chalcides ocellatus*			*Varanus exanthematicus*			*Agama impalearis*		
Lamina	Vertebral region	PS	S	C	PS	S	C	PS	S	C	PS	S	C
Anterior centrosynapophyseal lamina (ACYL)		-	x	~	~	x	~	x	~	~	x	x	-
Posterior centrosynapophyseal lamina (PCYL)		-	x	~	-	x	~	x	x	x	x	x	~
Prezygosynapophyseal lamina (PRYL)		-	-	-	~	-	~	~	x	~	-	-	~
Postzygosynapophyseal lamina (POYL)		-	x	-	-	-	-	-	-	-	-	-	-
Centroprezygapophyseal lamina (CPRL)		x	x	x	x	x	x	x	x	x	x	x	x
Interpreyzgapophyseal lamina (TPRL)		x	x	x	x	x	x	x	x	x	x	x	x
Spinoprezygapophyseal lamina (SPRL)		~	-	-	~	-	-	~	-	-	~	-	-
Postzygoprezygapophyseal lamina (PPRL)		-	-	-	x	x	x	~	-	x	x	~	~
Centropostzygapophyseal lamina (CPOL)		x	x	x	x	x	x	x	x	x	x	x	x
Interpostzygapophyseal lamina (TPOL)		-	-	-	-	-	-	-	-	~	-	-	-
Spinopostzygapophyseal lamina (SPOL)		x	x	x	x	x	x	x	x	x	x	x	x
Prespinal lamina (PRSL)		x	x	x	x	x	x	x	x	x	x	x	x
Postspinal lamina (POSL)		-	-	-	x	x	x	x	x	~	~	~	~

Presence in a vast majority of the elements is indicated by a “x”, absence in a vast majority of the elements by a dash, and ambiguity by a “~” (in the case of the SPRL, presence in the axis only is considered ambiguous here, otherwise it would be absent in all vertebral subregions, which is not true). Only skeletally mature individuals were considered here. Abb.: C, caudal vertebrae; PS, presacral vertebrae; S, sacral vertebrae.

**Table 3:** Vertebral laminae present in lacertid Squamata, showing occurrence in presacral, sacral, and caudal vertebrae.

Species		*Anatololacerta danfordi*			*Lacerta agilis*			*Lacerta bilineata*			*Lacerta strigata*			*Lacerta trilineata*			*Phoenicolacerta troodica*			*Podarcis muralis*			*Podarcis wagleriana*			*Takydromus* sp.			*Timon lepidus*			*Zootoca vivipara*		
Lamina	Vertebral region	PS	S	C	PS	S	C	PS	S	C	PS	S	C	PS	S	C	PS	S	C	PS	S	C	PS	S	C	PS	S	C	PS	S	C	PS	S	C
Anterior centrosynapophyseal lamina (ACYL)		-	~	~	-	x	~	-	x	~	-	x	~	-	~	~	-	x	~	-		(x)	-	x	~	-	~	~	-	x	~	-	x	~
Posterior centrosynapophyseal lamina (PCYL)		-	x	~	-	x	~	x	x	~	-	x	~	x	x	~	-	x	~	-		(x)	-	x	~	-	x	~	x	x	~	-	x	~
Prezygosynapophyseal lamina (PRYL)		-	~	~	-	~	-	-	~	~	-	x	~	-	x	~	-	x	~	-		(x)	-	x	~	-	x	~	-	x	~	-	x	~
Postzygosynapophyseal lamina (POYL)		-	x	-	-	x	-	-	-	-	-	-	-	-	~	-	-	x	x	-		(-)	-	x	-	-	-	-	-	x	-	-	-	-
Centroprezygapophyseal lamina (CPRL)		x	x	x	x	x	x	x	x	x	x	x	x	x	x	x	x	x	x	x		x	x	x	x	x	x	x	x	x	x	x	x	x
Interpreyzgapophyseal lamina (TPRL)		x	x	x	x	x	x	x	x	x	x	x	x	x	x	x	x	x	x	x		x	x	x	x	x	x	x	x	x	x	x	x	x
Spinoprezygapophyseal lamina (SPRL)		~	-	-	~	-	-	~	-	-	~	-	-	~	-	-	~	-	-	~		-	~	-	-	~	-	-	~	-	-	~	-	-
Postzygoprezygapophyseal lamina (PPRL)		x	-	~	x	-	~	x	-	~	x	x	~	x	-	~	~	-	~	x		~	x	-	~	~	-	~	x	~	~	x	-	~
Centropostzygapophyseal lamina (CPOL)		x	x	x	x	x	x	x	x	x	x	x	x	x	x	x	x	x	x	x		(x)	x	x	x	x	x	x	x	x	x	x	x	x
Interpostzygapophyseal lamina (TPOL)		-	-	~	-	-	~	-	-	~	-	-	~	-	-	~	-	-	~	-		(-)	-	-	~	-	-	~	-	-	~	-	-	~
Spinopostzygapophyseal lamina (SPOL)		x	x	x	x	x	x	x	x	x	x	x	x	x	x	x	x	x	x	x		(x)	x	x	x	x	x	x	x	x	x	x	x	x
Prespinal lamina (PRSL)		x	x	x	x	x	x	x	x	x	x	x	x	x	x	x	x	x	x	x		x	x	x	x	x	x	x	x	x	x	x	x	x
Postspinal lamina (POSL)		~	-	~	~	~	-	~	x	x	~	~	~	~	x	~	~	~	-	~		(-)	x	-	~	x	x	-	~	x	~	~	x	~

Presence in a vast majority of the elements is indicated by a “x”, absence in a vast majority of the elements by a dash, and ambiguity by a “~” (in the case of the SPRL, presence in the axis only is considered ambiguous here, otherwise it would be absent in all vertebral subregions, which is not true). Only skeletally mature individuals were considered here. The sole included specimen of *Podarcis muralis* does not preserve sacral vertebrae, and only few anterior, non-autotomic caudal vertebrae and a single anterior portion of an autotomic vertebra. No info on sacral vertebrae can thus be given for this taxon, and the information on caudal vertebrae in parentheses is limited to anterior, non-autotomic elements. Abb.: C, caudal vertebrae; PS, presacral vertebrae; S, sacral vertebrae.

The term lamina might seem exaggerated regarding some of the discussed structures in lizard vertebrae, where they are merely more than ridges. However, as mentioned above, laminae are not diagnosed by the fact that they are thin, plate-like structures, but by the fact that they connect two morphological landmarks, and do so throughout the entire column [21]. In fact, they can be interrupted, as is the case in the spinoprezygapophyseal laminae of most diplodocine sauropods [23,24]. For consistency reasons, it thus appears appropriate to stick to this proposed terminology. A comprehensive terminology allows for more straight forward comparisons between a large variety of vertebrates. Following this definition, some ridges in Lacertini vertebrae can be termed laminae.

A major adaptation for the nomenclature in lizards concerns the diapophyseal and parapophyseal laminae. Given that the diapophysis and the parapophysis fuse to form the synapophysis in presacral vertebrae [1], the original terms cannot be used in this group. Thus, diapophyseal and parapophyseal laminae have to be combined under the name of synapophyseal laminae. The other main groupings of laminae can be retained. Because the code SL is already taken by the spinal laminae [20], it cannot be applied to synapophyseal laminae. To avoid confusion, I therefore propose YL as general abbreviation for these types of laminae.

Another lamina that necessitates a name change is the epipophyseal-prezygapophyseal lamina (EPRL [21]). Several names have been proposed in saurischian dinosaurs for this lamina, which extends more or less horizontally across the lateral surface of the neural spine. Whereas it is clear that it connects to the prezygapophysis as principal landmark, the proposed names differ in the use of the postzygapophysis or the epipophysis as secondary landmark ([21] and references therein). The epipophysis is a ridge above the postzygapophyseal facets, which is well-known in dinosaurs, and has as well been identified in more distantly related archosauriforms (e.g. *Vancleavea campi* (figure 11A in [33]). Given that a similar structure in snakes is called pterapophysis (e.g. figure 1 in [34]), the use of the term epipophysis would be ambiguous. Furthermore, because these structures have never been described in Lacertini, I herein prefer the use of the postzygapophysis as secondary landmark, as previously proposed by Salgado et al. [35], creating the name postzygoprezygapophyseal lamina (PPRL).

## Occurrence and Serial Variation in Presacral Vertebrae

**Fig 1:**
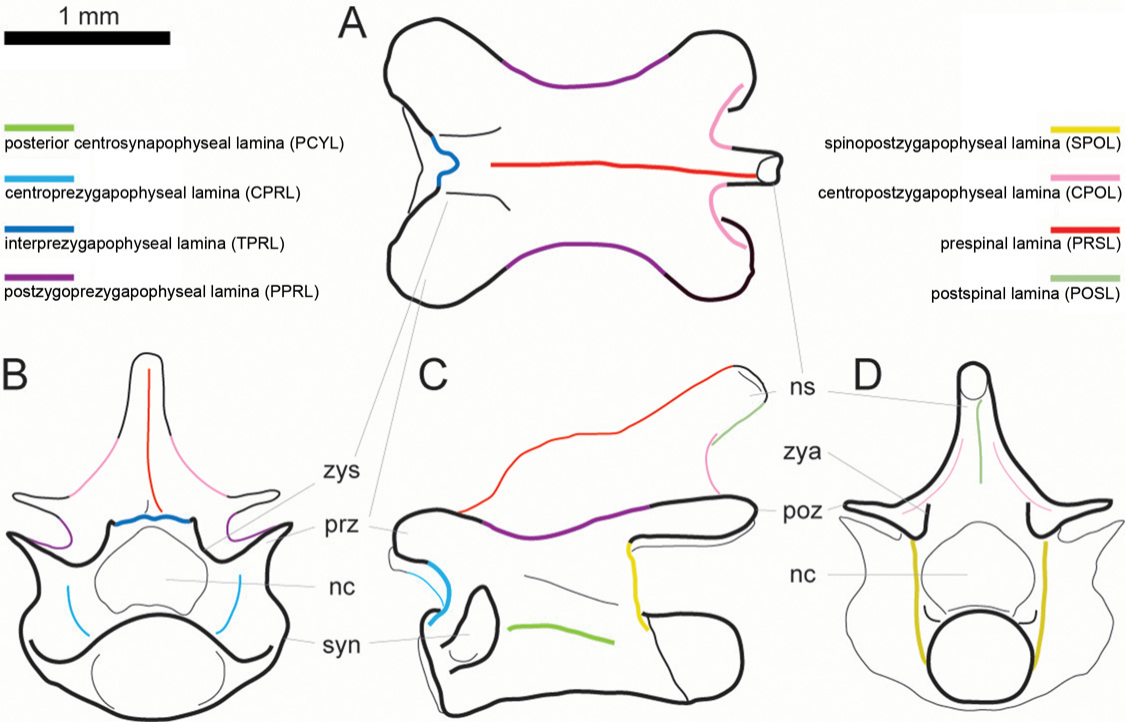
Laminae in presacral vertebrae of Lacertini. Hypothetical dorsal vertebra of a lacertid in dorsal (A), anterior (B), left lateral (C) and posterior (D) view (based on *Lacerta trilineata* MDHC 240), with the positions of the occurring laminae indicated. Scale bar = 1 mm. Abb.: nc, neural canal; ns, neural spine; poz, postzygapophysis; prz, prezygapophysis; syn, synapophysis; zya, zygantrum; zys, zygosphene.

The laminae observed in presacral vertebrae of Lacertini are the following (Table 3): posterior centrosynapophyseal lamina (PCYL), centroprezygapophyseal lamina (CPRL), interprezygapophyseal lamina (TPRL), postzygoprezygapophyseal lamina (PPRL), spinoprezygapophyseal lamina (SPRL), centropostzygapophyseal lamina (CPOL), spinopostzygapophyseal lamina (SPOL), prespinal lamina (PRSL), and postspinal lamina (POSL). Their position are shown in Fig 1.

### Posterior centrosynapophyseal lamina (PCYL)

**Fig 2:**
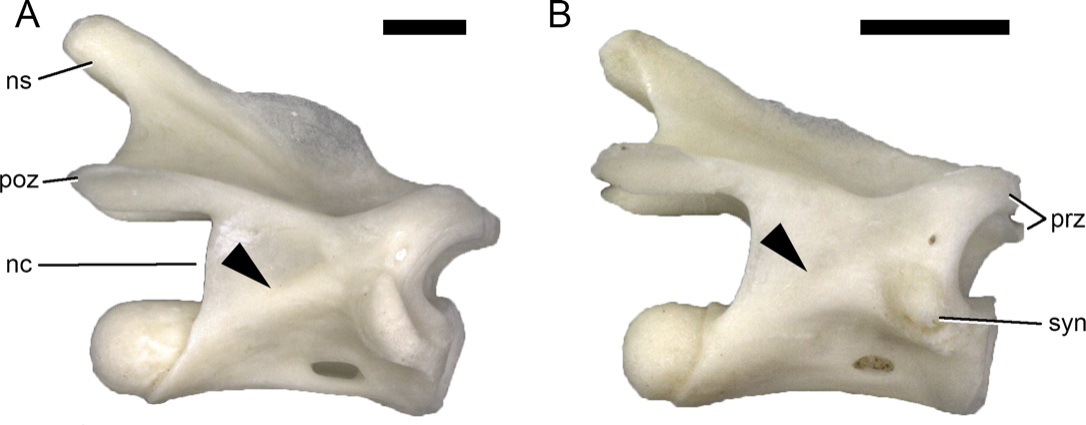
Size-related change in development of the PCYL. Dorsal vertebrae of *Lacerta trilineata* MDHC 240 (A) and *Lacerta strigata* MDHC 304 (B) in right lateral view. Arrowheads indicate the presence (A) and absence (B) of the PCYL in large (A) and small (B) lacertines. Vertebrae scaled to the same centrum length. Scale bars = 1 mm. Abb.: nc, neural canal; ns, neural spine; poz, postzygapophysis; prz, prezygapophysis; syn, synapophysis.

The PCYL extends from the synapophysis posteroventrally towards the dorsal margin of the centrum. It consistently occurs in *Lacerta bilineata*, *L*. *trilineata*, and *Timon lepidus*, where it is strongly developed. The PCYL is absent in small Lacertini (Fig 2). Of the non-lacertid squamates observed, the gecko *Tarentola* and the skink *Chalcides* did not have this lamina in presacral vertebrae. Medium- to large-sized lizards like *Agama* or *Varanus* have a well-developed PCYL.

Although the lateral surface of the neural arch pedicel is sometimes marked by a shallow convexity (e.g. in *Lacerta agilis*), such an indistinct convexity cannot be considered a lamina. Where it does not extend onto the centrum, it does not conform to the basic rules of vertebral laminae, which are supposed to connect two vertebral landmarks [20]. On the other hand, the continuous ridge between the dorsal portion of the synapophysis and the centrum of *L*. *bilineata* vertebrae is herein considered a PCYL. The lamina in *L*. *bilineata* slightly fades towards the centrum. The occurrence of this lamina might be size-related, as it seems restricted to larger forms within Lacertini, and intermediately large *L*. *bilineata* do have weakly developed PCYL. As in sauropod dinosaurs, they might thus increase vertebral stability without adding too much weight [20].

In the two specimens of *Lacerta bilineata*, where serial variation could be observed, the anterior-most and posterior-most presacral vertebrae do not have a PCYL. The number of elements without PCYL is variable (S1 Table).

### Centroprezygapophyseal lamina (CPRL)

The CPRL supports the prezygapophysis from below, and connects to the centrum. It occurs in all presacral vertebrae of all species studied herein (S2 Table).

**Fig 3:**
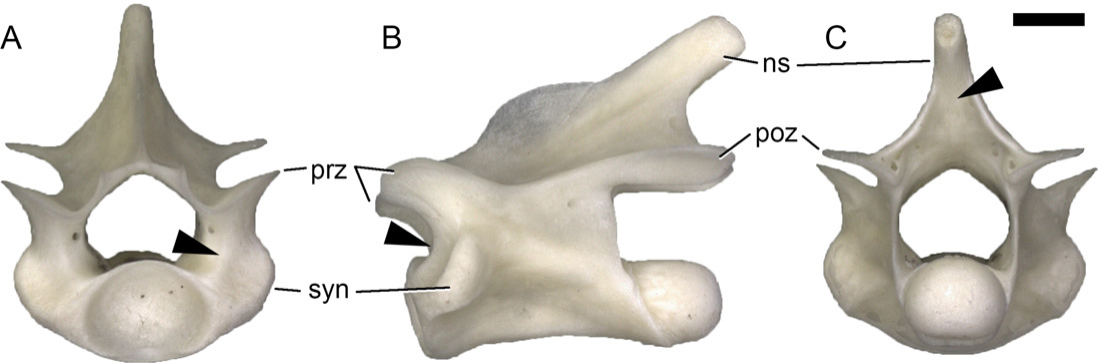
CPRL and POSL in lacertine presacral vertebrae. Dorsal vertebra of *Lacerta trilineata* MDHC 240, in anterior (A), left lateral (B) and posterior (C) view. Arrowheads indicate the CPRL with a crest (A, B), and the ridge-like POSL (C). Scale bar = 1 mm. Abb.: ns, neural spine; poz, postzygapophysis; prz, prezygapophysis; syn, synapophysis.

Generally, the transition from the internal surface of the neural canal onto the anterior surface of the synapophysis is a smooth curve, and does not form a distinct crest. Given that it still forms a connection between the centrum and the prezygapophysis that remains traceable throughout the entire column, it is here considered a lamina. However, in dorsal vertebrae of *Lacerta bilineata* and *L*. *trilineata*, for instance, the anterior surface of the synapophysis is transversely concave, and thus the CPRL becomes a dorsoventrally extending, anteriorly projecting crest below the prezygapophysis (Fig 3A and 3B). The transition from presacral vertebrae with smoothly rounded CPRL to the ones developing a crest is relatively smooth in *L*. *bilineata*, and largely corresponds to the cervico-dorsal transition. In *L*. *agilis*, a crest can sporadically occur, but there seems to be no consistent pattern. If it occurs, it is more pronounced ventrally than dorsally. In *Zootoca vivipara*, the crest can occur throughout the column, but is most expressed in the anterior dorsal vertebrae, whereas in *Takydromus*, the CPRL of presacral vertebrae do not form distinct crests.

### Interprezygapophyseal lamina (TPRL)

The TPRL connects the two prezygapophyses, and more precisely the zygosphenal facets (in the case of the Lacertini). They usually form the convex roof of the neural canal in members of the Lacertini. A TPRL occurs in all postaxial presacral vertebrae of all species studied herein (S3 Table), as well as in an incipient form in the axis of *Varanus exanthematicus* MDHC 335. In MDHC 335, the laminae connecting the prezygapophyses with the spine summit are interrupted by medially expanded bony shelves, which nearly contact each other at the midline to form the roof of the neural canal. These bony shelves are connected to the spine summit by two nearly parallel laminae enclosing a shallow fossa facing anteriorly. The laminae situated ventrally to the bony shelves are thus interpreted as incipient TPRL, because they nearly touch each other to form a continuous neural canal roof that remains separated from the spine summit. The laminae connecting the shelves with the summit can be interpreted as SPRL instead. *Varanus exanthematicus* MDHC 335 is thus the only specimen examined where both TPRL and SPRL occur in the axis.

**Fig 4:**
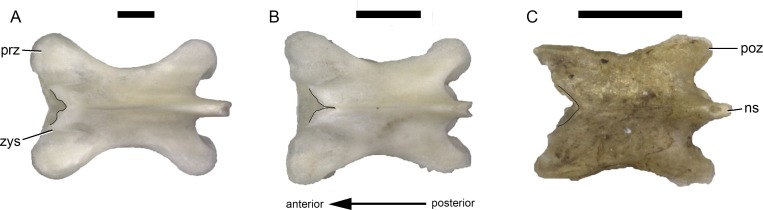
**Different morphologies of the TPRL in dorsal vertebrae of Lacertini (black lines): *Lacerta trilineata* MDHC 240 (A), *L*. *strigata* MDHC 304 (B), and *Phoenicolacerta troodica* MDHC 318 (C) in dorsal view.** Note the anterior projections in A, the narrow midline incision in B, and the regularly subtriangular TPRL in C. Vertebrae scaled to the same neural arch length. Scale bars = 1 mm. Abb.: ns, neural spine; poz, postzygapophysis; prz, prezygapophysis; zys, zygosphene.

In the lacertid axis, the prezygapophyses are connected with the spine through laminae, which only join at the spine summit. These cannot be considered a TPRL, but rather represent a SPRL. The TPRL, as interpreted herein, forms a continuous lamina between the prezygapophyses, which is detached from the neural spine summit. The TPRL can have various curvatures, which are highly variable throughout the column. However, it seems that in *Lacerta*, it generally develops a weak, centrally located, anterior projection in anterior cervical and most dorsal vertebrae, but it is more straight or even slightly concave in posterior cervical and some posterior-most dorsal elements. Some dorsal vertebrae have two anterior projections close to the midline, separated by a short, narrow incision (Fig 4). This incision could represent yet incompletely fused right and left halves of the neural arch, and thus be an ontogenetic feature. These have only been observed between positions PS 13 and 19 in *L*. *agilis*, but reach more anteriorly and posteriorly in *L*. *bilineata*. The apparent more restricted occurrence of the incision in *L*. *agilis* is probably due to the small sample size of articulated specimens, but might as well show that neural spine fusion first terminates in anterior and posterior presacral regions and happens only later in more central serial positions. In *Zootoca*, the TPRL has a wide V-shape in cervical and the posterior-most dorsal vertebrae, whereas it is more straight, or at least gently concave in most other dorsal elements. Only few vertebrae develop an anterior projection. In *Takydromus*, no vertebra was found with an anterior projection on the TPRL, but the majority of the elements had the midline incision potentially indicative of skeletal immaturity.

### Postzygoprezygapophyseal lamina (PPRL)

**Fig 5:**
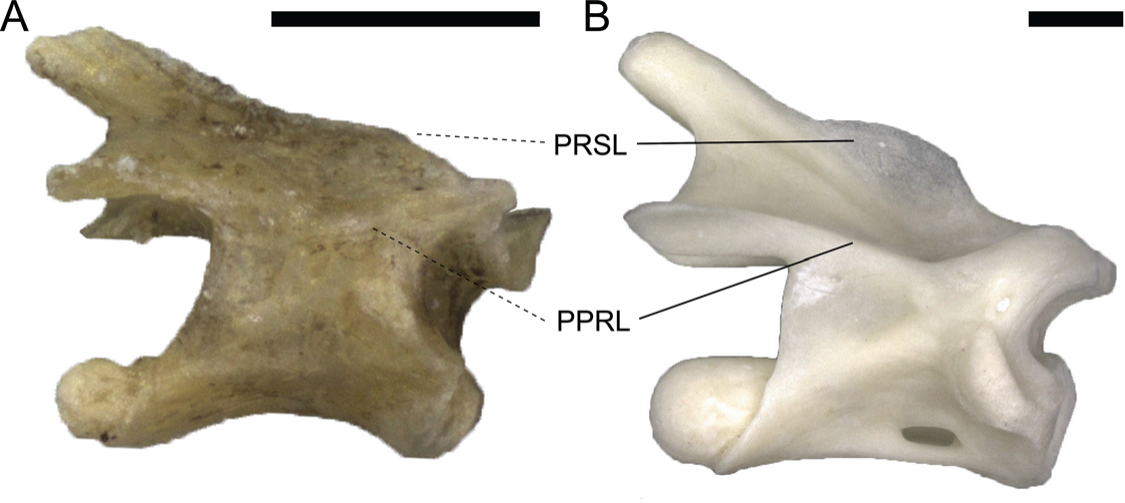
**Dorsal vertebrae of *Phoenicolacerta troodica* MDHC 318 (A) and *Lacerta trilineata* MDHC 240 (B) in right lateral view, showing reduced (dashed line, A) and well-developed (B) PPRLs and PRSLs.** Vertebrae scaled to the same centrum length. Scale bars = 1 mm. Abb.: PPRL, postzygoprezygapophyseal lamina; PRSL, prespinal lamina.

The PPRL connects the post- and prezygapophyses across the lateral surface of the neural spine, above the synapophysis. In nearly all observed lacertid taxa but *Phoenicolacerta troodica* and *Takydromus*, the PPRL occurs in all but the posterior-most dorsal vertebrae (S4 Table). An exception is a specimen of *Lacerta agilis*, where the axis does not bear a distinct PPRL. In *Takydromus*, only some anterior cervical and mid-dorsal elements could positively be scored for the presence of this lamina, and also in *P*. *troodica* it does not appear consistently throughout the column (Fig 5; a detailed assessment was not possible here due to the absence of articulated presacral columns, see Table 1). In the studied non-lacertid squamates, presacral vertebrae of the gecko *Tarentola* do not bear a PPRL. In the specimen of *Varanus*, the PPRL is only present in anterior cervical vertebrae, but instead marks caudal vertebrae of all subsections of the caudal column, whereas in lacertid caudal vertebrae, the occurrence of the PPRL is generally restricted to the non-autotomic and anterior autotomic elements.

The PPRL is always slightly inclined posterodorsally, although this inclination decreases in more posterior elements, following the greater elongation of these vertebrae compared to anterior cervical elements. The PPRL is very short in anterior cervical vertebrae, where the pre- and postzygapophyses nearly contact each other. It is most distinct in anterior and mid-dorsal vertebrae. Towards the posterior-most elements, the central area becomes gradually less distinct until the entire PPRL disappears in the last one or two dorsal vertebrae before the sacrum. The transition from present to absent, or relatively distinct ridge to gentle curve, is very gradual, and it is difficult to observe a clear border in the column. Where the PPRL is present, it is medially accompanied by a depression on the neural spine surface. In large forms like *Lacerta bilineata*, the neural arch pedicels below the PPRL are concave as well. Thus, in *L*. *bilineata*, the PPRL is more distinct than in smaller forms like *L*. *agilis* or *Zootoca vivipara*. However, the posterior-most one to two presacral vertebrae of *L*. *bilineata* still lose their PPRL. *Takydromus* has very faint to absent PPRL.

### Spinoprezygapophyseal lamina (SPRL)

**Fig 6:**
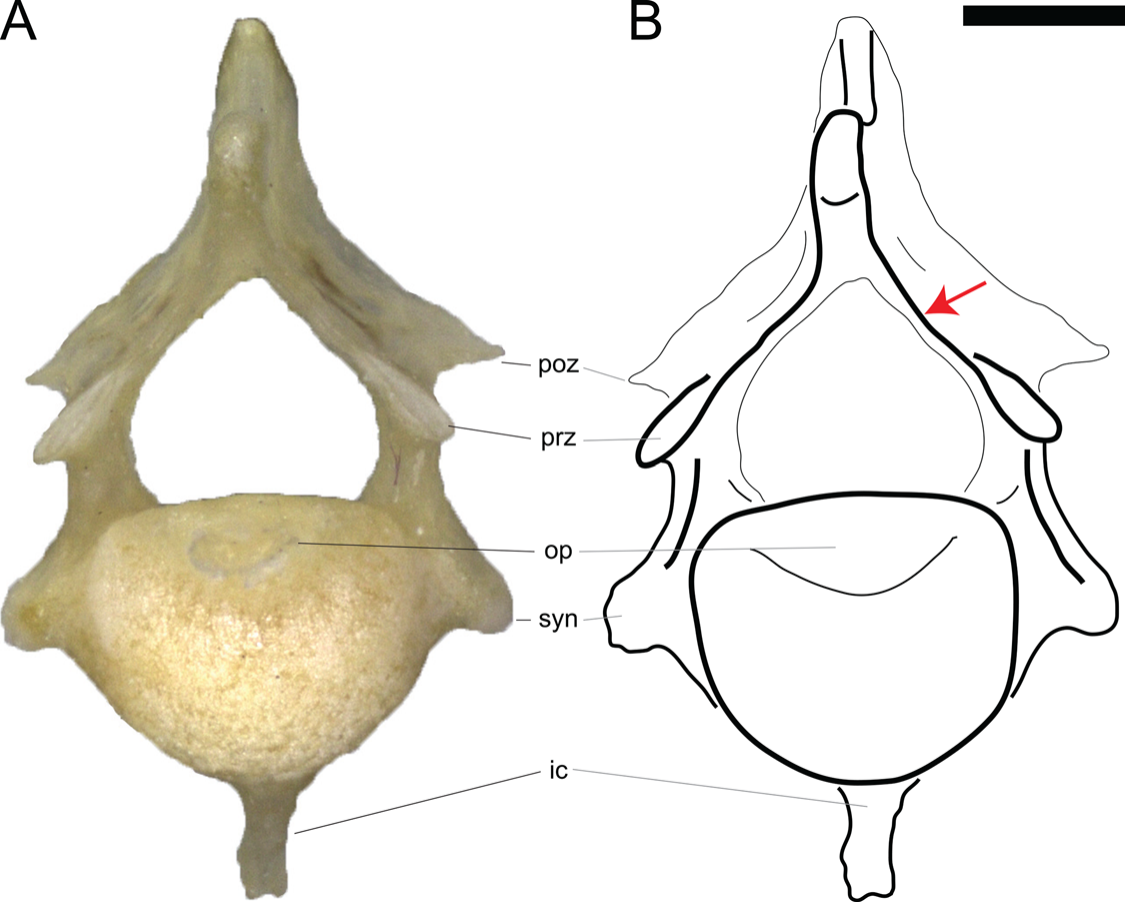
SPRL in the axis of *Lacerta bilineata* MDHC 15 in anterior view (arrow). Note that the left and right SPRL do not conjoin below the spine summit. Scale bar = 1 mm. Abb.: ic, intercentrum; op, odontoid process; poz, postzygapophysis; prz, prezygapophysis; syn, synapophysis.

The SPRL connects the prezygapophyses with the neural spine. They do not necessarily have to extend to the spine summit. The SPRL is different from the TPRL in that it rarely joins its counterpart on the midline, or if it does, the junction further involves the PRSL, relatively close to the spine summit [20]. In the squamates studied, the SPRL only occurs in the axis (S5 Table), but all observed species have an axial SPRL (Fig 6).

The axial SPRL might actually represent a serial homologue of the TPRL and PRSL as expressed in postaxial lacertine presacral vertebrae. The intermediate morphology in the axis of *Varanus exanthematicus* MDHC 335 indicates that the formation of the TPRL and PRSL in postaxial vertebrae could represent a case of lamina cutoff (sensu [21]), where the posterior displacement of the neural spine summit leads to the medial contact of the TPRL. Once the contact is completed, the SPRL might merge into a PRSL. However, since the two laminae in the lacertid axis apparently do not join medially, they should be considered to be SPRL.

### Centropostzygapophyseal lamina (CPOL)

The CPOL connects the dorsolateral corner of the rim around the articular condyle with the anteromedial corner of the postzygapophyseal facet. Hence, it does not contact the PPRL, which extends from the anterolateral corner of the facet anteriorly. The CPOL is consistently present among the studied lizards in all presacral vertebrae excluding the atlas (S6 Table).

**Fig 7:**
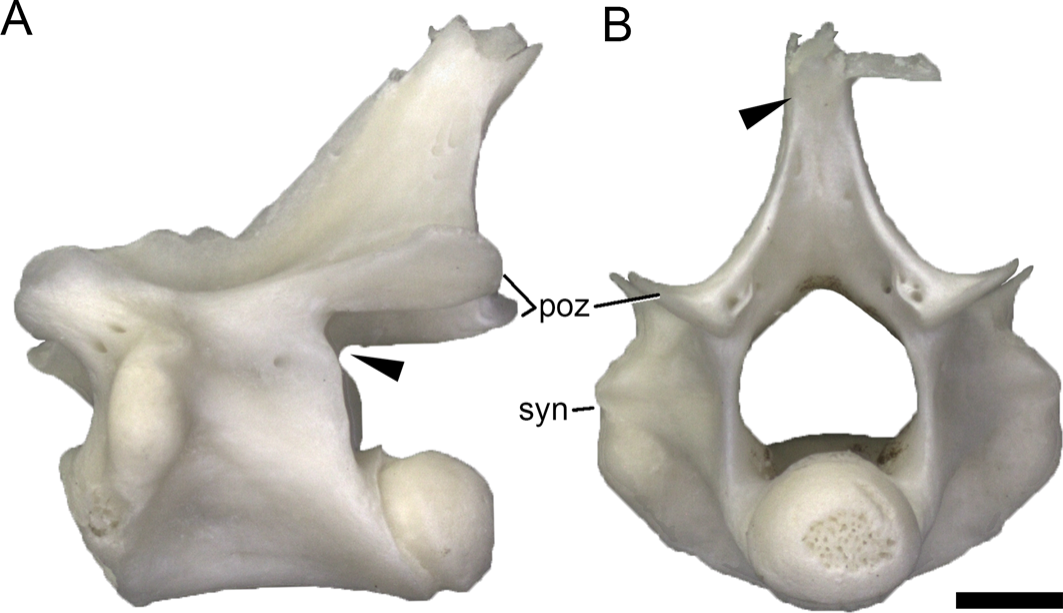
**Cervical vertebra of *Lacerta trilineata* MDHC 356 in left lateral (A) and posterior (B) view, showing dorsal incision of the CPOL (arrowhead, A), and the dorsal fading of the SPOL on the neural spine process (arrowhead, B).** The neural spine summit is damaged. Scale bar = 1 mm. Abb.: poz, postzygapophysis; syn, synapophysis.

The CPOL is generally straight to slightly concave in lateral view, and sometimes bears a weak posterior extension at varying levels. Given that the pedicel is usually somewhat anteriorly inclined, the CPOL extends anterodorsally in lateral view. In *Lacerta agilis*, this inclination decreases towards more posterior elements. Some specimens have CPOLs with a distinct dorsal incision, just below the contact with the postzygapophyseal facet (Fig 7A). These incisions occur in certain specimens of *L*. *bilineata* in anterior cervical and some dorsal vertebrae (where the distribution is irregular throughout the column). *Zootoca vivipara* has the incision only in anterior cervical elements, whereas it is restricted to mostly anterior dorsal vertebrae in *Takydromus*. The incision always leads into a short horizontal groove on the lateral surface of the neural arch pedicel.

### Spinopostzygapophyseal lamina (SPOL)

The SPOL extends from the posteromedial corner of the postzygapophyseal facet dorsomedially and forms the posterior margin of the neural spine in lateral view. However, its ventral base is located just lateral to the zygantrum. The right and left SPOL never contact each other dorsally. As the CPOL, the SPOL occurs in all species throughout the entire presacral column, except for the atlas (S7 Table).

The medial inclination of the SPOL depends upon the development of the neural spine process. Where this process is short, and oriented posteriorly (as e.g. in *Zootoca vivipara*), the SPOL approaches a horizontal orientation. In *Lacerta agilis*, the SPOL connects to the spine summit in the anterior-most cervical vertebrae and in mid- to posterior dorsal elements. In posterior cervical and anterior dorsal vertebrae of *L*. *agilis*, as well as in the entire presacral column of *L*. *bilineata*, the SPOL fades dorsally, before reaching the spine summit (Fig 7B).

### Prespinal lamina (PRSL)

The PRSL develops on the midline of the neural spine, connecting the spine summit with its base anteriorly. It is consistently present in all postaxial presacral vertebrae (S8 Table) of all species analyzed.

The axis has a longitudinal crest on the neural spine in a similar location as the more posterior elements, but this crest expands transversely, and appears to be an attachment site for ligaments throughout its length. It is therefore here considered to be an elongate spine summit, and not a PRSL. The PRSL nearly reaches the anterior edge of the TPRL in the presacral vertebrae of *Lacerta* and *Takydromus*, but is more posteriorly restricted in all but the posterior-most dorsal vertebrae of *Zootoca vivipara*. The PRSL usually develops an extremely thin, anterodorsally projecting blade of bone. This blade is of highly variable shape throughout the column (Fig 5). It is generally most developed in vertebrae with elevated neural spine processes, as in cervical and posterior dorsal vertebrae of *L*. *agilis*, and all presacral elements of *L*. *bilineata*. *Zootoca*, on the other hand, has only weakly developed blades. *Takydromus* presents the exception to the rule, in having relatively low spinal processes, but distinctly developed blades on the PRSL. In some vertebrae, the blades even exceed the height of the neural spine summit.

### Postspinal lamina (POSL)

The POSL is the equivalent lamina to the PRSL on the posterior surface of the neural spine, between the two SPOL. In this area, the neural spine is often slightly convex, in particular in cervical vertebrae, but it does not always develop a distinct ridge. Here, I only interpreted the POSL to be present, when a narrow, longitudinal midline ridge extends below the junction of the neural spine process with the two SPOLs (Fig 3C). Such a POSL does occur occasionally in all Lacertini studied, but without a clear pattern. The only consistent occurrence of the POSL throughout the Lacertini is in the posterior dorsal vertebrae (S9 Table), where it is always more distinct than in more anterior elements, if present. Among non-lacertid squamates, the POSL is absent in the gecko *Tarentola*, but marks vertebrae of all regions of the column of *Agama impalearis*, *Chalcides ocellatus*, and *Varanus exanthematicus*.

## Occurrence in Sacral and Caudal Vertebrae

**Fig 8:**
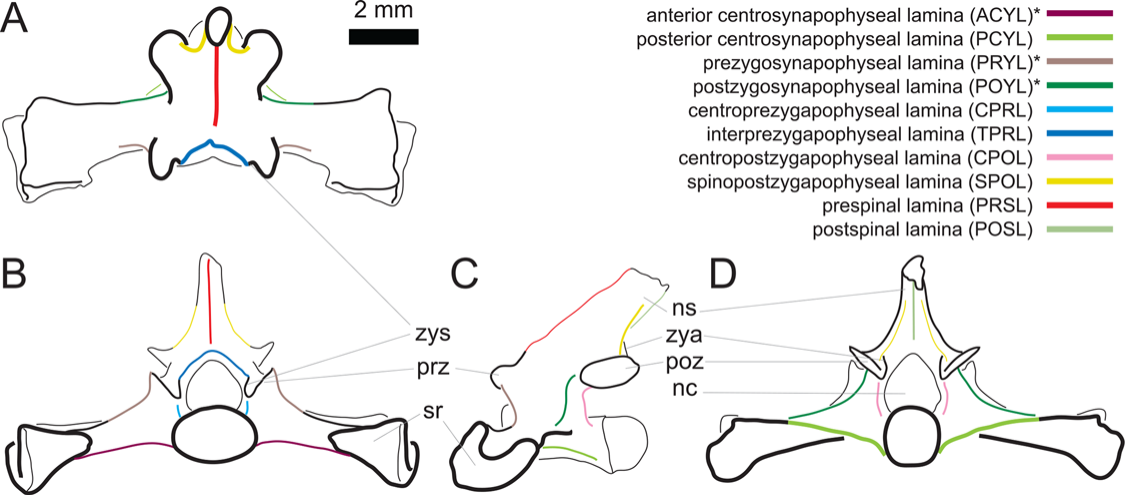
Laminae in sacral vertebrae of Lacertini. Hypothetical sacral vertebra 2 of a lacertid in dorsal (A), anterior (B), left lateral (C) and posterior (D) view (based on *Lacerta trilineata* MDHC 356), with the positions of the occurring laminae indicated. Asterisks mark the laminae only occurring in sacral and anterior caudal vertebrae of Lacertini. Scale bar = 2 mm. Abb.: nc, neural canal; ns, neural spine; poz, postzygapophysis; prz, prezygapophysis; sr, sacral rib; zya, zygantrum; zys, zygosphene.

Sacral vertebrae are highly modified to comply with functional needs of forming the connection to the hindlimb [1]. The stout sacral ribs are fused to the synapophysis, and form the connection to the ilium. In certain cases the distal ends of the sacral ribs of the two subsequent sacral vertebrae fuse [1]. The fused synapophyses and sacral ribs (sometimes called pleurapophyses [1,17]) are supported by varying combinations of four laminae, three of which never occur in presacral vertebrae in Lacertini. These four laminae comprise the anterior and posterior centrosynapophyseal laminae (ACYL and PCYL), and the pre- and postzygosynapophyseal laminae (PRYL and POYL; Fig 8).

The ACYL connects the anterior portion of the centrum with the anterodorsal margin of the synapophysis, and extends onto the sacral rib. Whereas the occurrence of the ACYL is restricted to sacral and caudal vertebrae in Lacertini, some non-lacertid squamates exhibit this lamina as well on presacral elements (e.g. *Agama impalearis* MDHC 275, *Chalcides ocellatus* MDHC 193, *Varanus exanthematicus* MDHC 335). In the presacral column of *C*. *ocellatus*, only cervical vertebrae have an ACYL. The cervical vertebrae of *C*. *ocellatus* have strongly laterally expanded synapophyses, such that the connection to the centrum forms a distinct ridge.

The PCYL persists onto the sacral and anterior caudal vertebrae (Tables 2, 3). As in presacral vertebrae, the PCYL is usually more robust in sacral and caudal vertebrae of large-sized specimens than in small ones.

The PRYL extends between the prezygapophysis and the anterior part of the synapophysis, and can coalesce with the ACYL in some cases. The PRYL does not occur in presacral elements of Lacertidae or *Agama impalearis*, but it is present in cervical vertebrae of *Chalcides ocellatus*, and on all presacral vertebrae of *Varanus exanthematicus*. No PRYL could be recognized in any vertebra of the gecko *Tarentola mauritanica*.

The POYL connects the postzygapophysis with the synapophysis (Fig 8). Both zygosynapophyseal laminae (PRYL and POYL) are more rarely expressed across lizards compared to the centrosynapophyseal laminae (ACYL and PCYL; Tables 2, 3). All four synapophyseal laminae mark varying numbers of anterior caudal vertebrae in different species.

**Fig 9:**
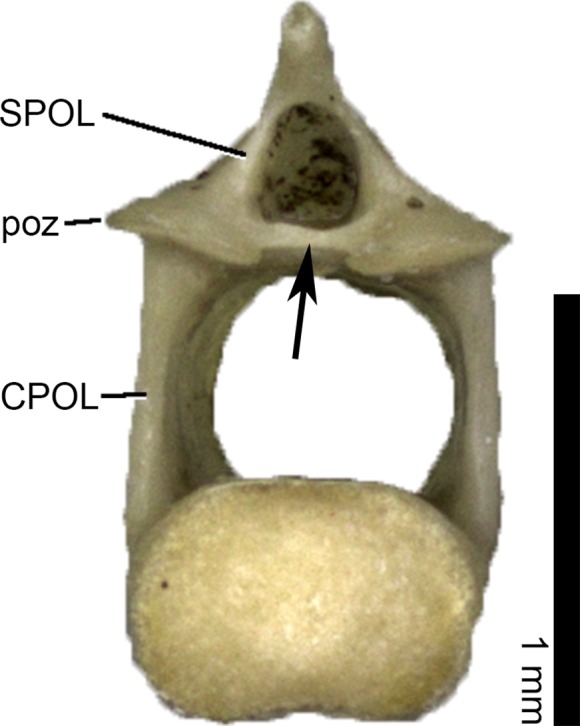
TPOL (arrow) in posterior autotomic caudal vertebra of *Podarcis wagleriana* MDHC 390 in posterior view. Scale bar = 1 mm. Abb.: CPOL, centropostzygapophyseal lamina; poz, postzygapophysis; SPOL, spinopostzygapophyseal lamina.

In posterior autotomic caudal vertebrae of certain taxa, an interpostzygapophyseal lamina (TPOL) occurs (Fig 9). The only lamina that is never present in any sacral or caudal vertebra is the SPRL.

### Serial variation in sacral vertebrae of Lacertini

The ACYL is restricted to the anterior sacral vertebra in *Anatololacerta danfordi* and *Lacerta trilineata*. In *L*. *agilis*, *Podarcis wagleriana*, *Timon lepidus*, *Zootoca vivipara*, and *Phoenicolacerta troodica*, the ACYL is more distinct in the anterior elements, but still present in the posterior sacral vertebra. In *Takydromus* sp., the ACYL only occurs in the second sacral element. The PCYL increases in robustness from anterior to posterior sacral elements in *A*. *danfordi*, *Podarcis wagleriana*, and *Phoenicolacerta troodica*, whereas the opposite is the case in *L*. *agilis*, *L*. *bilineata*, *L*. *trilineata*, *Takydromus* sp., and *Timon lepidus*. The PRYL is restricted to the first sacral vertebra of *A*. *danfordi*, *L*. *agilis*, and *L*. *bilineata*. In *Phoenicolacerta troodica*, *Podarcis wagleriana*, *Takydromus* sp., *Timon lepidus*, and *Zootoca vivipara*, the PRYL occurs on both sacral elements, but is more strongly developed in sacral vertebra 1. The POYL is restricted to the first sacral element in some specimens of *L*. *trilineata*. It occurs in both vertebrae in *Phoenicolacerta troodica*, *Podarcis wagleriana*, and *Timon lepidus*, but in these species, it is less developed in the posterior sacral element.

As in presacral vertebrae, the CPRL of the anterior sacral element develops a crest in some specimens of *L*. *agilis*, *L*. *bilineata*, *L*. *strigata*, *L*. *trilineata*, *Timon lepidus*, and *Zootoca vivipara*. The PPRL is restricted to the second sacral vertebra of *Timon lepidus*. The POSL is restricted to the second sacral vertebra of *L*. *strigata* and *Phoenicolacerta troodica*.

### Serial variation in caudal vertebrae of Lacertini

Laminae generally decrease in robustness throughout the caudal column, as long as they do not form basic vertebral structures like the neural arch, as is the case with the CPRL, TPRL, CPOL, and the SPOL. An exception to the weakening in posterior direction is the PRSL, which often forms an anterodorsally projecting spur, or large sagittal plates (e.g. in *Takydromus* sp.) in middle to posterior caudal vertebrae.

Different laminae persist throughout varying subregions of the caudal column in different taxa. For instance, the ACYL is only present on the anterior, non-autotomic vertebrae of *Anatololacerta danfordi*, but persists throughout this subregion and disappears within the anterior autotomic sequence in *Lacerta agilis*, *L*. *bilineata*, *L*. *strigata*, *L*. *trilineata*, *Takydromus* sp., *Timon lepidus*, and *Zootoca vivipara*. The PCYL persists onto autotomic elements in all species studied here. The PRYL disappears within anterior, non-autotomic caudal vertebrae in *A*. *danfordi*, *L*. *strigata*, *L*. *trilineata*, *Podarcis wagleriana*, and *T*. *lepidus*. In *Takydromus* sp., the PRYL only occurs on the first caudal vertebra, in Z*ootoca vivipara* it marks the first two caudal elements, whereas in *L*. *agilis*, and *L*. *bilineata* it persists into the autotomic subregion. The PPRL disappears on the last non-autotomic vertebra in *L*. *strigata*, *L*. *trilineata*, *Podarcis muralis*, *Podarcis wagleriana*, *Timon lepidus*, and *Zootoca vivipara*, whereas it persists onto the first autotomic vertebra in *L*. *agilis* and *L*. *bilineata*. In species in which the POSL occurs, it only marks the first few caudal vertebrae.

## Ontogenetic Changes and Taxonomic Utility

Ontogenetic changes in vertebral lamination were proposed in sauropod dinosaurs, where skeletally very immature individuals appear to have weakly developed laminae [25,32,36]. A general increase in vertebral lamination through ontogeny was herein observed in Lacertini as well.

As mentioned in the Material section, three specimens could be assigned to different ontogenetic stages based on vertebral fusion patterns. The most immature stage is represented by *Lacerta bilineata* MDHC 73, followed by *L*. *bilineata* MDHC 48, and finally *Phoenicolacerta troodica* MDHC 319. The TPRL, CPOL, and SPOL occur in all ontogenetic stages, because they form the neural canal in Lacertini. The same accounts for the SPRL in the axis. Similarly, the presence and development of the PPRL does not appear to change through ontogeny.

**Fig 10:**
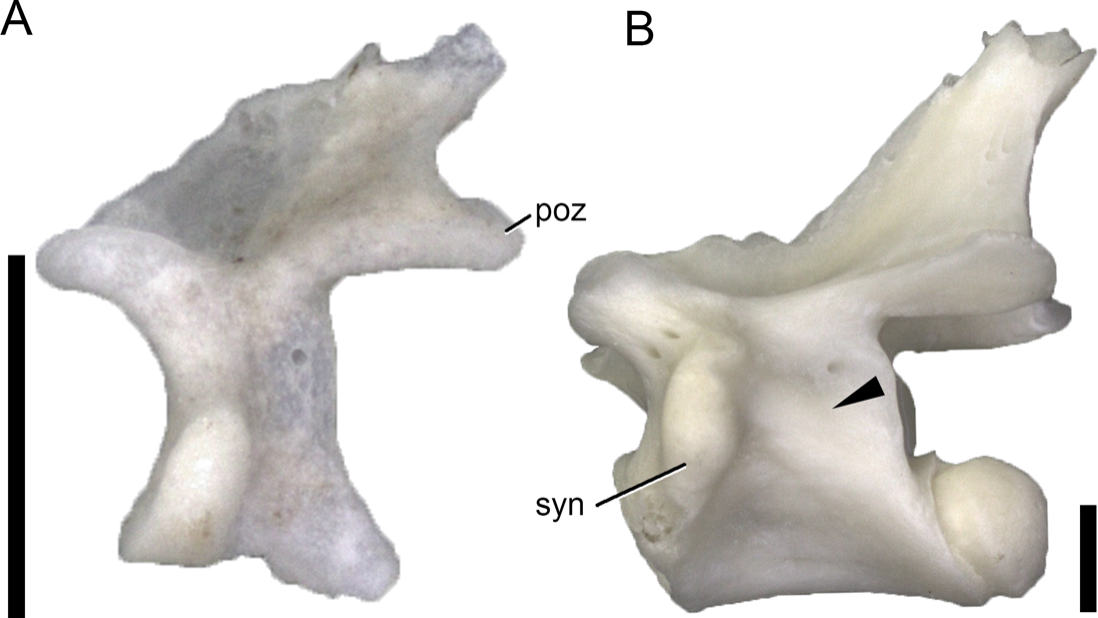
Size-related change of development of the PCYL. Cervical vertebrae of the skeletally immature *Lacerta bilineata* MDHC 48 (separate left neural arch, A), and the mature *L*. *trilineata* MDHC 356 in left lateral view. Note the well-developed (arrowhead, B) PCYL in the large, mature specimen, in contrast to the small, immature specimen, where no PCYL occurs (A). The neural spine summit of MDHC 356 is damaged. Vertebrae are scaled to the same neural arch length. Scale bars = 1 mm. Abb.: poz, postzygapophysis; syn, synapophysis.

Ontogenetic changes were observed in the PCYL, CPRL, PRSL, and POSL. Both skeletally immature specimens of *L*. *bilineata* do not have a PCYL, indicating that this lamina develops late in ontogeny in this species. Alternatively, given that small-sized adult Lacertini generally do not show a PCYL, the development might simply be size-related, and occur once juvenile *L*. *bilineata* specimens reach the size, where the formation of a PCYL becomes a structural necessity (Fig 10).

The development of the crest on the CPRL appears to be more complicated. Whereas this crest often occurs in adult *Lacerta bilineata*, the two juvenile *L*. *bilineata* only rarely have crested CPRLs, and the *Phoenicolacerta troodica* only shows them in completely fused vertebrae. This indicates a late onset of development of this crest, which is unrelated to size.

Being located on the neural arch midline, the PRSL and POSL can only develop after the fusion of the right and left halves of the arch. However, as soon as fusion is complete, the distribution of these laminae approximates the one in skeletally mature individuals.

Some occurrences of laminae are not obviously correlated with ontogenetic changes nor do they seem to be size-related. For instance, the occurrence of the PPRL in general and its distribution in sacral vertebrae appears taxonomically useful: it is present in the relatively small *Lacerta strigata*, but absent in presacral vertebrae of the large-sized *Varanus exanthematicus*, and restricted to the second sacral element in the medium-sized *Timon lepidus* (Tables 2, 3). The occurrence of the TPOL in posterior caudal elements is restricted to lacertids of varying body size and *V*. *exanthematicus*, whereas it is absent in the gecko *Tarentola mauritanica* and the medium-sized *Agama impalearis*. The absence of the ACYL and PRYL in presacral vertebrae of Lacertini might represent a feature distinguishing them from non-lacertid lizards, where some observed taxa have presacral ACYL and PRYL. Whereas it is not in the scope of this paper to identify diagnostic traits of certain clades, these observations show that the occurrence of particular vertebral laminae should be tested for taxonomic significance, and possibly included in phylogenetic analyses of the clade. The nomenclature proposed herein is a first step to facilitate the recognition of potentially diagnosing features, and their translation into phylogenetic character statements.

## Conclusions

Vertebral lamination has been recognized and identified in lizards. A terminology generally used in archosauriforms was adapted to lacertine morphology, and proved to be useful outside the clade for which it was initially proposed. Probably due to the small size of lizards, only 13 of about 20 laminae proposed in saurischian dinosaurs [20,21] occur in their vertebrae.

As in sauropod dinosaurs, lamination increases during ontogeny in lacertine lizards. Some laminae, like the posterior centrosynapophyseal lamina (which is equivalent to the posterior centrodiapophyseal, and posterior centroparapophyseal laminae in archosauriforms), only appear in large-sized individuals.

Differences in occurrence and variability in serial variation along the presacral column of lizards might prove taxonomically valid in future. In particular, the ACYL, PRYL, PPRL and the TPOL appear to be valuable candidates to help distinguish certain species or clades. The adaptation of the nomenclature will allow for more detailed descriptions in future, which will be needed to identify diagnostic osteological features and recognize morphological phylogenetic characters in lizards.

## Supporting Information

S1 Table Posterior centrosynapophyseal lamina (PCYL), serial variation in presacral vertebrae of Lacertini. Boxes represent the vertebrae in the column, including the atlas. Filled boxes indicate presence of the lamina in the respective vertebrae, whereas a dash stands for absence. Only the seven specimens with articulated vertebral column could be assessed.
(PDF)

S2 Table Centroprezygapophyseal lamina (CPRL), serial variation in presacral vertebrae of Lacertini. Boxes represent the vertebrae in the column, including the atlas. Filled boxes indicate presence of the lamina in the respective vertebrae, whereas a dash stands for absence. Only the seven specimens with articulated vertebral column could be assessed.
(PDF)

S3 Table Interprezygapophyseal lamina (TPRL), serial variation in presacral vertebrae of Lacertini. Boxes represent the vertebrae in the column, including the atlas. Filled boxes indicate presence of the lamina in the respective vertebrae, whereas a dash stands for absence. Only the seven specimens with articulated vertebral column could be assessed.
(PDF)

S4 Table Postzygoprezygapophyseal lamina (PPRL), serial variation in presacral vertebrae of Lacertini. Boxes represent the vertebrae in the column, including the atlas. Filled boxes indicate presence of the lamina in the respective vertebrae, whereas a dash stands for absence. Only the seven specimens with articulated vertebral column could be assessed.
(PDF)

S5 Table Spinoprezygapophyseal lamina (SPRL), serial variation in presacral vertebrae of Lacertini. Boxes represent the vertebrae in the column, including the atlas. Filled boxes indicate presence of the lamina in the respective vertebrae, whereas a dash stands for absence. Only the seven specimens with articulated vertebral column could be assessed.
(PDF)

S6 Table Centropostzygapophyseal lamina (CPOL), serial variation in presacral vertebrae of Lacertini. Boxes represent the vertebrae in the column, including the atlas. Filled boxes indicate presence of the lamina in the respective vertebrae, whereas a dash stands for absence. Only the seven specimens with articulated vertebral column could be assessed.
(PDF)

S7 Table Spinopostzygapophyseal lamina (SPOL), serial variation in presacral vertebrae of Lacertini. Boxes represent the vertebrae in the column, including the atlas. Filled boxes indicate presence of the lamina in the respective vertebrae, whereas a dash stands for absence. Only the seven specimens with articulated vertebral column could be assessed.
(PDF)

S8 Table Prespinal lamina (PRSL), serial variation in presacral vertebrae of Lacertini. Boxes represent the vertebrae in the column, including the atlas. Filled boxes indicate presence of the lamina in the respective vertebrae, whereas a dash stands for absence. Only the seven specimens with articulated vertebral column could be assessed.
(PDF)

S9 Table Postspinal lamina (POSL), serial variation in presacral vertebrae of Lacertini. Boxes represent the vertebrae in the column, including the atlas. Filled boxes indicate presence of the lamina in the respective vertebrae, whereas a dash stands for absence. Only the seven specimens with articulated vertebral column could be assessed.
(PDF)
