# Supplementary material for: Nomenclature of Vertebral Laminae in Lizards, with Comments on Ontogenetic and Serial Variation in Lacertini (Squamata, Lacertidae)
Source: PLoS One. 2016 Feb 23;11(2):e0149445. doi: 10.1371/journal.pone.0149445 (PMC4764367; doi:10.1371/journal.pone.0149445)
Supplement: S6 Table — Boxes represent the vertebrae in the column, including the atlas. Filled boxes indicate presence of the lamina in the respective vertebrae, whereas a dash stands for absence. Only the seven specimens with articulated vertebral column could be assessed. (PDF) [file pone.0149445.s006.pdf]

S6 Table. Centropostzygapophyseal lamina (CPOL), serial variation in presacral vertebrae of Lacertini. Boxes represent the vertebrae in the column, including the atlas. Filled boxes indicate presence of the lamina in the respective vertebrae, whereas a dash stands for absence. Only the seven specimens with articulated vertebral column could be assessed.

Species	Specimen	Cervical Vertebrae	Dorsal Vertebrae
Lacerta agilis	MDHC 176	– ■■■■■■■	■■■■■■■■■■■■■■■■■■■■■
Lacerta agilis	MDHC 177	– ■■■■■■■	■■■■■■■■■■■■■■■■■■■■
Lacerta agilis	MDHC 178	– ■■■■■■■	■■■■■■■■■■■■■■■■■■■
Lacerta bilineata	MDHC 15	– ■■■■■■■	■■■■■■■■■■■■■■■■■■■
Lacerta bilineata	MDHC 77	– ■■■■■■■	■■■■■■■■■■■■■■■■■■■
Zootoca vivipara	MDHC 179	– ■■■■■■■	■■■■■■■■■■■■■■■■■■■■
Takydromus sp.	MDHC 151	– ■■■■■■■	■■■■■■■■■■■■■■■■■■■
