# Supplementary material for: Nomenclature of Vertebral Laminae in Lizards, with Comments on Ontogenetic and Serial Variation in Lacertini (Squamata, Lacertidae)
Source: PLoS One. 2016 Feb 23;11(2):e0149445. doi: 10.1371/journal.pone.0149445 (PMC4764367; doi:10.1371/journal.pone.0149445)
Supplement: S9 Table — Boxes represent the vertebrae in the column, including the atlas. Filled boxes indicate presence of the lamina in the respective vertebrae, whereas a dash stands for absence. Only the seven specimens with articulated vertebral column could be assessed. (PDF) [file pone.0149445.s009.pdf]

**S9 Table. Postspinal lamina (POSL), serial variation in presacral vertebrae of Lacertini.**
Boxes represent the vertebrae in the column, including the atlas. Filled boxes indicate presence of the lamina in the respective vertebrae, whereas a dash stands for absence. Only the seven specimens with articulated vertebral column could be assessed.

| Species | Specimen | Cervical Vertebrae | Dorsal Vertebrae |
|---|---|---|---|
| *Lacerta agilis* | MDHC 176 | – – – – – – – – | – – – – – – – – – – – – – – – – – – – – – |
| *Lacerta agilis* | MDHC 177 | – – – – – – – – | – – – – – – – – ■ – – ■ – – ■ ■ ■ – – ■ |
| *Lacerta agilis* | MDHC 178 | – – ■ ■ – – – – | – – ■ – – – – – ■ – – – – ■ ■ ■ ■ ■ ■ |
| *Lacerta bilineata* | MDHC 15 | – ■ ■ ■ ■ – – – | – ■ ■ ■ ■ ■ ■ ■ ■ ■ ■ ■ ■ ■ ■ ■ ■ – |
| *Lacerta bilineata* | MDHC 77 | – ■ ■ – – – – – | – – – – – ■ – – – – – – – ■ ■ ■ ■ ■ ■ |
| *Zootoca vivipara* | MDHC 179 | – – – – ■ – – – | – – – ■ – – – – ■ ■ ■ – – ■ ■ ■ ■ ■ ■ – |
| *Takydromus* sp. | MDHC 151 | – – – – ■ ■ ■ ■ | – ■ ■ ■ ■ ■ ■ ■ ■ ■ – – ■ ■ ■ ■ ■ ■ ■ |
